# *Blastocystis* across humans, animals and the environment in rural Türkiye, and relationships with the human intestinal microbiome

**DOI:** 10.3389/fmicb.2025.1665966

**Published:** 2025-10-20

**Authors:** Eylem Akdur-Öztürk, Yaseen Majid Salman Al-Adilee, William Edwards, Eleni Gentekaki, Anastasios D. Tsaousis, Funda Dogruman-Al

**Affiliations:** ^1^Department of Medical Parasitology, Faculty of Medicine, Çukurova University, Adana, Türkiye; ^2^School of Natural Sciences, University of Kent, Canterbury, United Kingdom; ^3^Department of Medical Microbiology, College of Medicine, Ninevah University, Mosul, Iraq; ^4^Department of Veterinary Medicine, University of Nicosia School of Veterinary Medicine, Nicosia, Cyprus; ^5^Division of Parasitology, Department of Microbiology, Gazi University, Ankara, Türkiye

**Keywords:** *Blastocystis*, gut microbiome, transmission dynamics, rural, microbial eukaryotes, Türkiye

## Abstract

*Blastocystis* is a globally prevalent intestinal protist commonly found in humans and animals, yet its role in health and disease remains ambiguous. This is a cross-sectional study of *Blastocystis* in rural Türkiye, examining 124 human, 305 livestock (cattle, sheep, goats), and 40 environmental samples using culture/microscopy, qPCR, and sequencing. We further explored associations between *Blastocystis* and population parameters, along with gut microbiota profiles. Using a combination of sequencing and microscopy, the overall prevalence was high, at 76.6% in humans, 71%–78% in livestock, and 38% in environmental samples. Subtypes ST1–ST4 were detected in humans, with ST3 being most frequent. Livestock harbored ST10 predominantly, with goats showing high carriage of ST24. Several subtypes (e.g., ST25, ST26) were recorded in livestock for the first time in Türkiye. Body mass index (BMI) was significantly associated with *Blastocystis* colonization, with lean individuals having higher carriage. Contrary to other studies, individuals with ST4 exhibited reduced bacterial diversity and altered microbial composition, suggesting subtype-specific interactions. By combining parasitology, microbiome, and environmental analysis, this study offers an overview of *Blastocystis* diversity and distribution in rural Türkiye. This work provides a foundation for future integrative research approaches to explore the ecological role of *Blastocystis* and its subtypes, potential health implications, and interactions with other microbes in rural and global contexts.

## Introduction

*Blastocystis* is a common intestinal protist found in humans and various animals, including mammals, birds, and reptiles. It is one of the two stramenopiles known to inhabit the human gut (Hublin et al., 2021; Nguyen et al., 2023). Despite its widespread presence, its role in health and disease remains unclear, making it a subject of ongoing research (Centers for Disease Control and Prevention [CDC], 2025).

The organism exists in multiple forms–vacuolar, granular, amoeboid, cystic, and less commonly avacuolar and multivacuolar –transmitting through the fecal-oral route via cysts shed in feces (Hublin et al., 2021; Tan, 2008). *Blastocys*tis is estimated to colonize nearly one billion people globally (Stensvold and Clark, 2020) with prevalence varying from 5%–20% in developed regions to over 30% in developing areas (Tan, 2008; Khorshidvand et al., 2021). Studies of human populations across diverse geographic regions suggest that *Blastocystis* colonization is associated with distinct gut microbial profiles and higher levels of microbial diversity. It has also been associated with increases in the abundance of beneficial bacterial taxa such as *Ruminococcaceae* and *Prevotella* (Audebert et al., 2016; Beghini et al., 2017; Tito et al., 2019). This is in contrast to the reduced microbial diversity typically observed in individuals with gastrointestinal diseases such as inflammatory bowel disease. Morever, *Blastocys*tis has been reported to be associated with healthy dietary patterns, and lower rates of obesity, cardiometabolic risk, and mortality (Piperni et al., 2024). These findings suggest that *Blastocystis* may be indicative of healthy gut microbiota though the underlying mechanisms of how this might be achieved remain unknown (Audebert et al., 2016; Beghini et al., 2017; Nieves-Ramírez et al., 2018; Kodio et al., 2019; Tito et al., 2019; Alzate et al., 2020; Castañeda et al., 2020; Even et al., 2021).

Based on the diversity of *SSU* rRNA, at least 44 *Blastocystis* subtypes (STs) have been identified. Among these, 16 STs, including ST1–ST10, ST12, ST14, ST16, ST23, ST35, and ST41, have been identified in humans, with ST1–ST4 being the most frequently reported (McCain et al., 2023; Koehler et al., 2024; Santin et al., 2024). In Türkiye, *Blastocystis* prevalence ranged from 2.1 to 51% across studies, with ST3 (47.9%) as the dominant subtype (Malatyalı et al., 2023). Studies on livestock (i.e., cattle, sheep, water buffaloes, and chickens), and companion animals (i.e., dogs, cats, and horses) together with those on environmental sources indicate carriage rates of 3.65% to over 60%, but transmission dynamics remain poorly understood (Onder et al., 2021; Tavur and Önder, 2022).

Recent data suggest that there is interplay of body mass index (BMI) and *Blastocystis* colonization, with several studies reporting higher *Blastocystis* presence in lean individuals (Beghini et al., 2017; Asnicar et al., 2021; Malatyali et al., 2021; Matovelle et al., 2022), while one study found a higher *Blastocystis* prevalence in obese individuals, though the obese sample size was small (Jinatham et al., 2021). Other studies reported prevalence exceeding 40% in obese populations but the absence of lean controls limits interpretation (Caudet et al., 2022a,b).

Zoonotic transmission studies suggest that certain subtypes may spread between humans and animals, but it remains unclear whether these same strains establish colonization or are merely transient (Tsaousis et al., 2025; Abdo et al., 2021; Ruang-areerate et al., 2021; Rudzińska et al., 2022). Recent studies have reported molecular detection of *Blastocystis* in soil (Jinatham et al., 2021; Blackburn et al., 2024), with certain subtypes shared between human and environmental samples (Jinatham et al., 2021). These findings suggest soil as a transmission route, adding another layer of complexity to its transmission. Hence, an integrative approach that considers humans, animals, and the environment, is essential for shedding light to the organism’s epidemiology (Tsaousis et al., 2024). The first study to investigate *Blastocystis* occurrence in this context took place in a rural community in Thailand (Jinatham et al., 2021). Nevertheless studies from diverse regions are essential to assess potential geographic or rural/urban differences. To address this gap, we conducted a study in a rural area of Türkiye. We aimed to explore diversity, distribution, and possible transmission dynamics of *Blastocysti*s in humans, livestock (cattle, sheep, goats), and environmental samples from Seyhan Dam Lake in Kırıklı village using microscopy and molecular methods. Moreover, we investigated the relationship between *Blastocystis* colonization and human gut microbiota composition.

## Materials and methods

This is a cross-sectional study conducted in Kırıklı village located in the Seyhan Dam Lake basin, Adana, between October and November 2023. Human, animal (cattle, sheep, and goats), and environmental (water and mud) samples were collected and analyzed using microscopy and molecular methods.

### Ethics statement and research permissions

The ethics committee of Çukurova University approved this study for sample collection (approval number 49/135) and microbiome analysis (approval number 39/147). Ethical rules were according to the Declaration of Helsinki. All participants were informed about the nature of the project. Signed consent was obtained from the participants and the parents of the child participants.

For the environmental samples, research permission was obtained from the Adana Governorate and Karaisalı District Directorate of Agriculture and Forestry for the use of dam lake materials (approval number E-12757666-140.03).

### Study area

**FIGURE 1:**
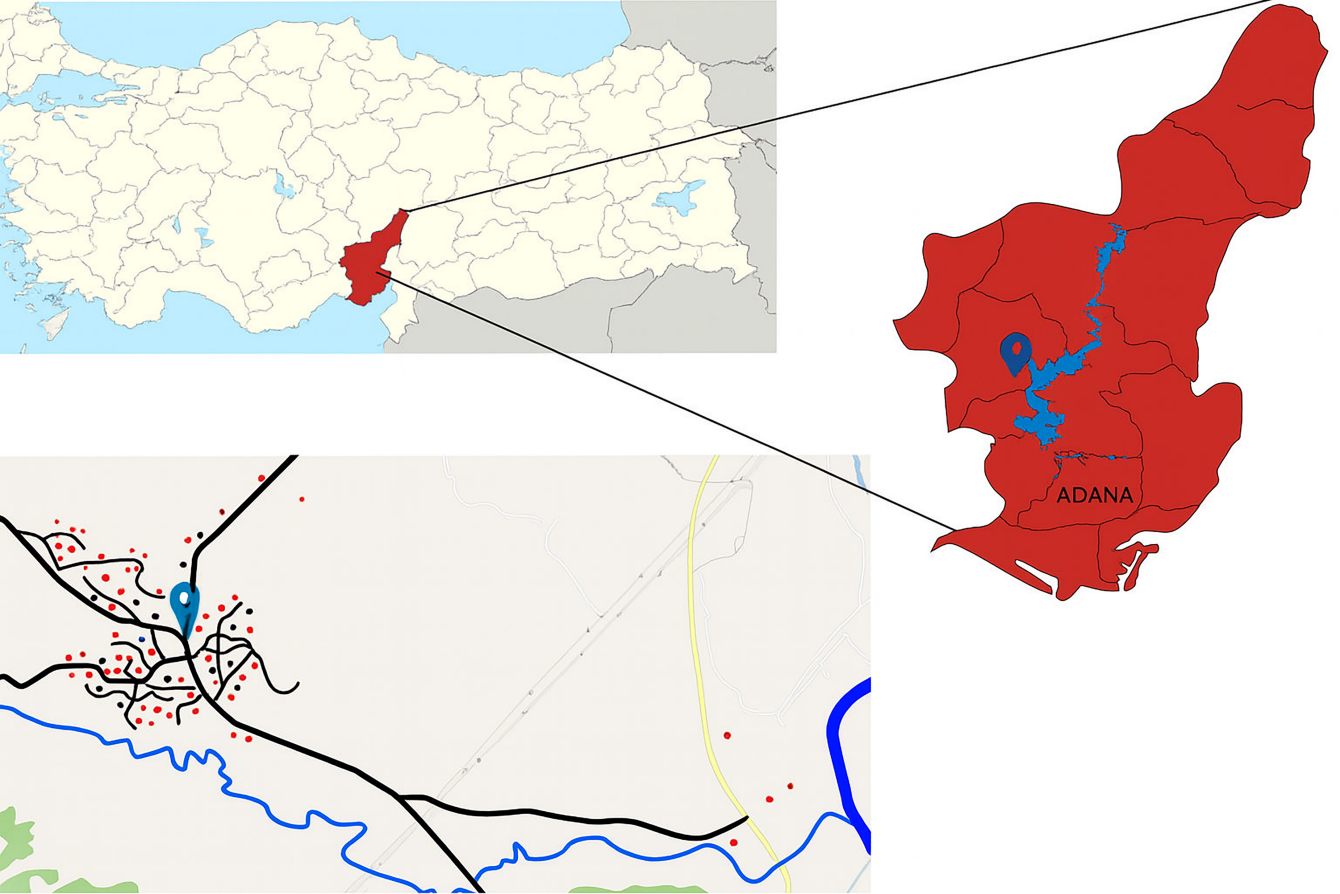
Map of Türkiye (top left), highlighting the sampling province in red. The bottom image shows sampling areas in Kırıklı Village (village’s location marked with a pin). Red circles represent households where both human and animal samples were collected; black circles represent households where only human samples were collected.

This study was conducted in Kırıklı, a village with a population of 582, located in Karaisalı district of Adana province, Türkiye (37 °10′N, 35 °14′E), 35 km away from the city center (Figure 1). The village is located within the Mediterranean climate zone, characterized by hot, dry summers and mild, rainy winters. The settlement, whose residents derive their living from farming and animal husbandry, is surrounded by agricultural lands, forest patches, grazing areas and tributaries of the Seyhan Dam Lake. Seasonal animal movements are common in the areas surrounding the village. The Seyhan Dam was constructed around 70 years ago and is located 12 km from the Kırıklı village (Supplementary Figure 1). Seyhan Dam Lake passes through the border of the village in the study area and is used to irrigate agricultural areas and animals. The water level recedes during November and December. Thus, the area becomes accessible for recreational activities, such as picnic and camping. It is also used as pasture for grazing animals, where they defecate. The area fills with water again in April and May due to snowmelt. This region was selected as a model site for the One Health approach due to its geographical location, climate, proximity to the Seyhan Lake Dam (used for livestock watering and field irrigation) substantial animal populations, rich pastures, and a population primarily engaged in agriculture and animal husbandry. Furthermore, the area has the potential to represent the human-animal-environment cycle of intestinal parasites.

### Sample collection

Human, animal (cattle, sheep and goats) and environmental samples were collected and preserved in a 1:2 ratio DNA/RNA Shield™ (ZymoResearch, Freiburg, Germany) until further analysis.

#### Human fecal samples

The “purposive sampling method,” commonly utilized in biological research and modified accordingly, was used (Işıklar, 2018) to determine the number of human samples for this study. All participants appeared to be healthy, with no cases of diarrhea or bloody stool. Samples were randomly collected from 124 participants across 64 households. Of these, 90 participants belonged to households actively engaged in animal husbandry, while the other 34 participants were from households without animals and reported not owning any animals for at least 6 months. These two groups were evaluated based on whether they were engaged in animal husbandry or not. There were no cases of diarrhea or bloody stool. Each participant was provided with a labeled, sterile fecal collection container.

#### Animal fecal samples

A total of 305 fecal samples were collected from 89 cattle, 151 sheep, and 65 goats belonging to the individuals participating in the study. The number of samples for each animal was calculated using the “proportional stratified sampling method” (Işıklar, 2018). Therefore, the number of animals sampled per household varied according to the household’s animal population. To minimize environmental contamination, samples were collected by direct observation of the animals’ defecation during morning feeding, grooming, or rest, so that only a single sample was collected from each animal. Individual samples were collected in pre-labeled containers.

#### Environmental samples

Twenty-four dam lake water and 16 mud samples were collected directly from the closest parts of the Seyhan Dam lake, where human activities (picnics, camping, fishing, etc.) and animal grazing occurred and from areas irrigated for livestock and agricultural use. Water samples were collected from the dam lake, its tributaries, and from standing water in 1-liter sterile containers. These were left to stand on a flat surface at room temperature overnight (Jinatham et al., 2021), and the supernatant was drained until 50 ml of sediment was left with a manual drainage system. Then, the sediment was transferred to 15 ml tubes and centrifuged at 500 *g* for 10 min, the supernatant was drained, and the sediment remaining at the bottom was preserved in a 1:2 ratio DNA/RNA shield until further analysis. Mud samples were taken from the mentioned locations and directly preserved in a 1:2 ratio DNA/RNA shield until further analysis.

### Screening of *Blastocystis*

#### Culture

Approximately 200 mg were taken from all fecal samples and inoculated into 2 ml of Jones’ medium containing 10% horse serum (Stensvold et al., 2007; Sarzhanov et al., 2021). Similarly, sediment obtained from water and mud samples processed as described in the Environmental Samples section was also inoculated into the medium. After 48–72 h of incubation at 37 °C, culture samples were examined using a light microscope (Supplementary Figure 2) to determine whether *Blastocystis* was present.

#### Genomic DNA Extraction

DNA extraction was performed by taking 200 μl of a thoroughly vortexed DNA/RNA shield-sample mixture, and the PureLink™ Microbiome DNA Purification Kit (Thermo Fisher Scientific, Carlsbad, CA, USA) was used according to the manufacturer’s protocol.

#### qPCR (Real-time PCR)

*Blastocystis* was identified using specific primer sequences (BL18SPPF1 _5′-AGTAGTCATACGCTCGTCTCAAA-3′ and BL 18SR2PP 5 ′-TCTTCGTTACCCGTTACTGC-3′) to amplify a conserved region of SSU rRNA gene (330 bp) by qPCR (Poirier et al., 2011). A negative control (nuclease-free water) and a positive control (genomic DNA of *Blastocystis*) were used in every qPCR run. The reaction mixtures (10 μl) contained 5 μl of Luna Taq Universal (New England Biolabs, Ipswich, MA, USA), 0.5 μl of a 10 μM primer pair, and 2 μl of template DNA. qPCR protocol included; pre-denaturation step: 95 °C for 5 min; followed by 49 cycles of denaturation step: 95 °C for 5 s, annealing step, 68 °C for 10 s, extension step: 72 °C for 15 s and final extension at 72 °C for 10 min. Reactions were set up in 96-well plates in a CFX96 Touch Real-Time PCR Detection System (Bio-Rad, United States).

#### Polymerase chain reaction and phylogenetic analysis

Nested PCR was performed on samples positive for *Blastocystis* by either culturing (microscopy) or qPCR methods. The primer oligonucleotide sequences of nested PCR are detailed in Supplementary Table 1. First and second-round PCR protocols were set up as follows: pre-denaturation step: 95 °C for 5 min; 30 cycles of: denaturation step: 94 °C for 1 min; annealing step: 59 °C and 50 °C (first and second-round PCR, respectively) for 1 min; extension step: 72 °C for 1 min and a final extension step: 72 °C for 10 min. DNA sequencing was performed by using internal primers for each second-round PCR-positive sample (Cologne, Germany).

The sequences obtained by Sanger sequencing were manually inspected and used as queries to perform BLAST searches for comparisons with reference gene sequences in the National Center for Biotechnology Information (NCBI). For the phylogenetic analysis, a dataset spanning *Blastocystis* diversity, as well as newly derived sequences, was constructed. To avoid redundancy, groups of highly similar sequences (% divergence <98%) were collapsed and only one or two representatives were included in the dataset. Sequences were aligned using MUSCLE v5. Ambiguous positions were removed using Trimal v1.4. Maximum likelihood analysis was performed using IQTREE (Nguyen et al., 2015).

#### Microbiome sequencing

High-throughput amplicon sequencing was outsourced to Novogene, following a modified version of the protocol described by Caporaso et al., 2011. One nanogram of extracted DNA was used, fragmented, and adapted for paired-end sequencing. The 16S rRNA gene was amplified using the primer pair 515F (GTGCCAGCMGCCGCGGTAA) and 907R (CCGTCAATTCCTTTGAGTTT), which targets the V3-V4 hypervariable region. Sequencing was performed on the Illumina NovaSeq platform.

Raw sequencing reads were processed using the Lotus2 pipeline (Özkurt et al., 2022). The workflow included several key steps: chimera detection and removal were conducted using Minimap2 (Li, 2018), which was also employed to identify and exclude off-target human DNA reads by performing a BLAST search against the Genome Reference Consortium Human Build 38.p14 (0 contaminated samples detected). The trimmed reads were then clustered into Amplicon Sequence Variants (ASVs) with a maximum of one nucleotide difference, using the Divisive Amplicon Denoising Algorithm 2 (DADA2) (Callahan et al., 2016). ASVs were taxonomically classified through BLAST searches against the GreenGenes2 (GG2) database (DeSantis et al., 2006).

### Statistical analysis

The data obtained in this study was statistically analyzed using IBM Statistical Package for the Social Sciences (SPSS) version 29 software. A global chi-square test was used to assess whether the distributions of gender and age groups (0–18 years, 19–39 years, 40–59 years, and 60 years and above) differed significantly (*p* < 0.05). For microbiome analysis, the Shapiro-Wilk test was used to assess the normality of data distribution. Normally distributed data were analyzed using ANOVA, followed by the Tukey’s HSD test for pairwise comparisons. For non-normally distributed data, the Kruskal-Wallis test was applied, followed by the Dunn test (with Bonferroni adjustment) for multiple comparisons.

Statistical analyses and data visualization were performed using R Studio 4.2.3. To account for variations in sequencing depth, data were first rarefied to 60,000 reads based on the species accumulation curve (Supplementary Figure 1). Rarefaction resulted in the exclusion of one sample. The relative abundance of each genus was then calculated for each sample, and a heatmap was generated to visualize the results. Alpha diversity was assessed using diversity indices, including Shannon and Simpson, and richness estimators including Chao1 and observed taxa, implemented in the Phyloseq package. These indices were compared between *Blastocystis*-positive and *Blastocystis*-negative samples. To visualize microbiome composition, compositional bar plots were generated using the Microbiome package, including only taxa representing more than 1% of total reads. Principal Coordinate Analysis (PCoA) based on Bray-Curtis dissimilarity was used to visualize differences in microbial community structure between *Blastocystis*-positive and *Blastocystis*-negative samples. Samples were plotted using Bray-Curtis dissimilarity matrices. PERMANOVA (Anderson, 2017) was used to test for statistical significance of group differences. Linear Discriminant Analysis Effect Size (LEfSe) (Segata et al., 2011) was applied to identify potential biomarkers discriminating *Blastocystis*-positive from *Blastocystis*-negative samples based on relative abundance profiles.

## Results

### Demographic characteristics of the human participants

Of the 124 participants in the study, 58 (46.8%) were female, and 66 (53.2%) were male, with an average age of 44.7 (range between 6 and 82). Of the participants, 90 out of 124 (72.6%) are actively involved in animal husbandry, and 34 (27.4%) stated being involved in caring for any animals for at least the last 6 months. Additionally, all participants declared that they drank tap water. Body mass index (BMI) was calculated for all participants who were 19 years of age or older (*n*:109) (Geifman and Rubin, 2011). Ninety-five (87.2%) of the individuals participating in the study had a BMI above 25 and were classified as overweight or obese. The remaining participants were classified as having normal weight (18.5–24.9). There were no individuals in the study who were extremely underweight (BMI < 18.5). The distribution of sociodemographic characteristics of the participants according to the presence of *Blastocystis* is shown in Table 1. A more detailed table can be found in Supplementary Table 1.

**TABLE 1:** Distribution of sociodemographic characteristics of participants according to the presence of *Blastocystis*.

Characteristics		Positive (*n*%)	Negative (*n*%)	Total (%)	*p* value
Gender	Female	40 (69.0%)	18 (31.0%)	58 (46.8%)	*p* ≈ 0.095 χ2≈ 2.7842
	Male	54 (81.8%)	12 (18.2%)	66 (53.2%)	
Age group	0–18	10 (66.7%)	5 (33.3%)	15 (12.1%)	*p* ≈ 0.79 χ^2^ = 1.048
	19–39	21 (80.8%)	5 (19.2%)	26 (21.0%)	
	40–59	45 (76.3%)	14 (23.7%)	59 (47.6%)	
	60 and above	18 (75.0%)	6 (25.0%)	24 (19.4%)	
BMI*	18.5–24.9	14 (100.0%)	0 (0.0%)	14 (12.8%)	***p* ≈ 0.032** χ^2^ = 6.892
	25.0–29.9	28 (66.7%)	14 (33.3%)	42 (38.5%)	
	≥30.0	42 (79.2%)	11 (20.8%)	53 (48.6%)	
Animal husbandry	Yes	65 (72.2%)	25 (27.8%)	90 (72.6%)	*p* ≈ 0.129 χ^2^ = 2.297
	No	29 (85.3%)	5 (14.7%)	34 (27.4%)	

*Body mass index (BMI) is calculated for ages 19 and above. Bold values indicate statistical significance (*p* < 0.05).

#### *Blastocystis* occurrence

In this study, all sample types were investigated for the presence of *Blastocystis* using culture and qPCR. A sample was considered positive when *Blastocystis* was seen microscopically (Supplementary Figure 2) in culture or when a sequence was obtained (qPCR product or PCR product). Hence, samples positive only for microscopy were included in the prevalence calculation but not subtyping. In all cases, the vacuolar form was predominantly observed microscopically and occasionally granular and amoeboid forms. In human samples, culture positivity was 70.1% (87/124). Regarding qPCR, 111/124 samples yielded a band of the expected size, however of these only 36 samples were successfully sequenced and subtyped. 28 samples were positive by both microscopy and molecular methods. Combining the two approaches, the overall positivity rate in humans in this study was 76.6% (95/124). Positivity in the 0–18 age group was 66.7% (10/15), 80.8% (21/26) in the 19–39 group, 76.3% (45/59) in the 40–59 group and 75% (18/24) in the over 60 group. Statistical analysis showed a significant association between BMI and *Blastocystis* positivity (*p* < 0.05). Though *Blastocysti***s** was more prevalent among individuals with normal BMI no statistical test was performed at the subgroup level. Global chi square tests revealed no statistically significant relationships between *Blastocystis* positivity and other demographic variables such as gender, occupation, and age groups (*p* > 0.05).

In animals, 66% (200/304) were culture positive. More specifically, 65.2% (58/89) of cattle were culture positive, 66.2% (100/151) of sheep, and 64.6% (42/65) of goats. Using qPCR, 100% of the samples showed a band of the expected size. Of these, 77 were successfully sequenced and subtyped (24 cattle, 35 sheep, and 17 goats). Combining the two approaches, the positivity rate was as 71% (63/89) in cattle, 73% (110/151) in sheep, and 78% (50/64) in goats.

The environmental samples showed 37.5% (15/40) positivity solely through culture. Eleven of these were water samples, while four were mud. None of the environmental samples yielded *Blastocystis* sequences.

#### *Blastocystis* subtypes

In total, 112 sequences were subtyped from humans (*n* = 36), cattle (*n* = 24), sheep (*n* = 35) and goat (*n* = 17). In multiple instances the chromoatograms showed evidence of mixed infections, as indicated by multiple peaks. To assign subtype the criteria below were followed: reasonably good quality sequence of over 300 bp and over 98% similarity in GenBank. Subtypes were identified using a combination of phylogeny and blast against GenBank and the curated database pubmlst^1^ and phylogeny (Supplementary Figure 3). In the phylogenetic analysis all subtypes were monophyletic and the new sequences placed within known subtype clades. For 18 sequences, the methodologies did not agree. In pubmlst, they were identified as belonging to one of the subtypes previously comprising ST10 (i.e., ST23, ST42-ST44), perhaps due to the short length of the sequences. In the phylogenetic tree, these placed within the clade comprising ST10 in the past, but jumped within the clade depending on taxon sampling. Of these, 13 were from cattle, three from sheep and two from goats. These sequences were not assigned a subtype, only the clade with which they cluster.

**FIGURE 2:**
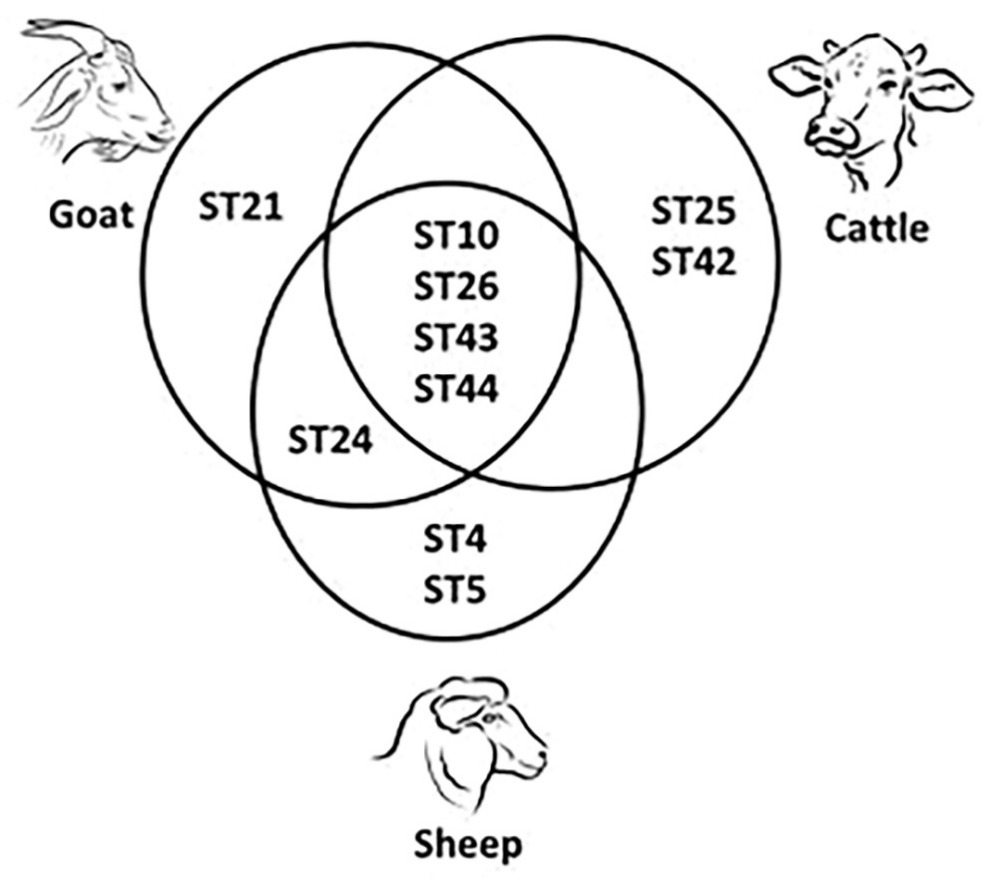
Shared and unique *Blastocystis* subtypes (ST) detected in cattle, sheep and goats.

In total, 13 subtypes were identified: ST1, ST2, ST3, ST4, ST5, ST10, ST21, ST24, ST25, ST26, ST42, ST43, and ST44. In humans, four subtypes were detected, namely ST1, ST2, ST3, and ST4. The most abundant subtype was ST3 (33%, 12/36), followed by ST1, and ST2 (each at 31%, 11/36) and ST4 (6%, 2/36). In animals, ten subtypes were detected: ST4, ST5, ST10, ST21, ST24, ST25, ST26, ST42, ST43, and ST44. In cattle, ST10 (*n* = 8), ST25 (*n* = 2), ST26 (*n* = 3), ST42 (*n* = 5), ST43 (*n* = 1), and ST44 (*n* = 5) were identified. In sheep, ST4 (*n* = 1), ST5 (*n* = 2), ST10 (*n* = 17), ST24 (*n* = 5), ST26 (*n* = 4), ST43 (*n* = 3), and ST44 (*n* = 3) were detected. Goat samples were positive for ST10 (*n* = 5), ST21 (*n* = 2), ST24 (*n* = 7), ST26 (*n* = 1), ST43 (*n* = 1), and ST44 (*n* = 1). Table 2 showed the distribution of *Blastocystis* subtypes among human and animal host. ST10 was the most abundant in cattle and sheep, while in goats ST24 was. ST10, ST26, ST43, and ST44 were shared by all three ruminant species (Figure 2). ST25 and ST42 were detected only in cattle, while ST21 was detected only in goats. ST24 was detected in goats and sheep, but not in cattle.

**TABLE 2:** Distribution of **Blastocystis** subtypes among human and animal hosts.

Host	ST1 n (%)	ST2 n (%)	ST3 n (%)	ST4 n (%)	ST5 n (%)	ST10 n (%)	ST21 n (%)	ST24 n (%)	ST25 n (%)	ST26 n (%)	ST42 n (%)	ST43 n (%)	ST44 n (%)	Total n
Human	11 31%	11 31%	12 33%	2 6%										36
Cattle						8 33.3%			2 8.3%	3 12.5%	5 20.8%	1 4.1%	5 20.8%	24
Sheep				1 2.9%	2 5.7%	17 48.6%		5 14.3%		4 11.4%		3 8.6%	3 8.6%	35
Goat						5 29.4%	2 11.8%	7 41.2%		1 5.9%		1 5.9%	1 5.9%	17
Total	11	11	12	3	2	30	2	12	2	8	5	5	9	112

At the household level, subtype analysis was performed on more than one individual from five households, and no common subtype (ST) was identified among individuals within the same household. However, common subtypes were reported among livestock within the same household.

### Microbiome analysis

**FIGURE 3:**
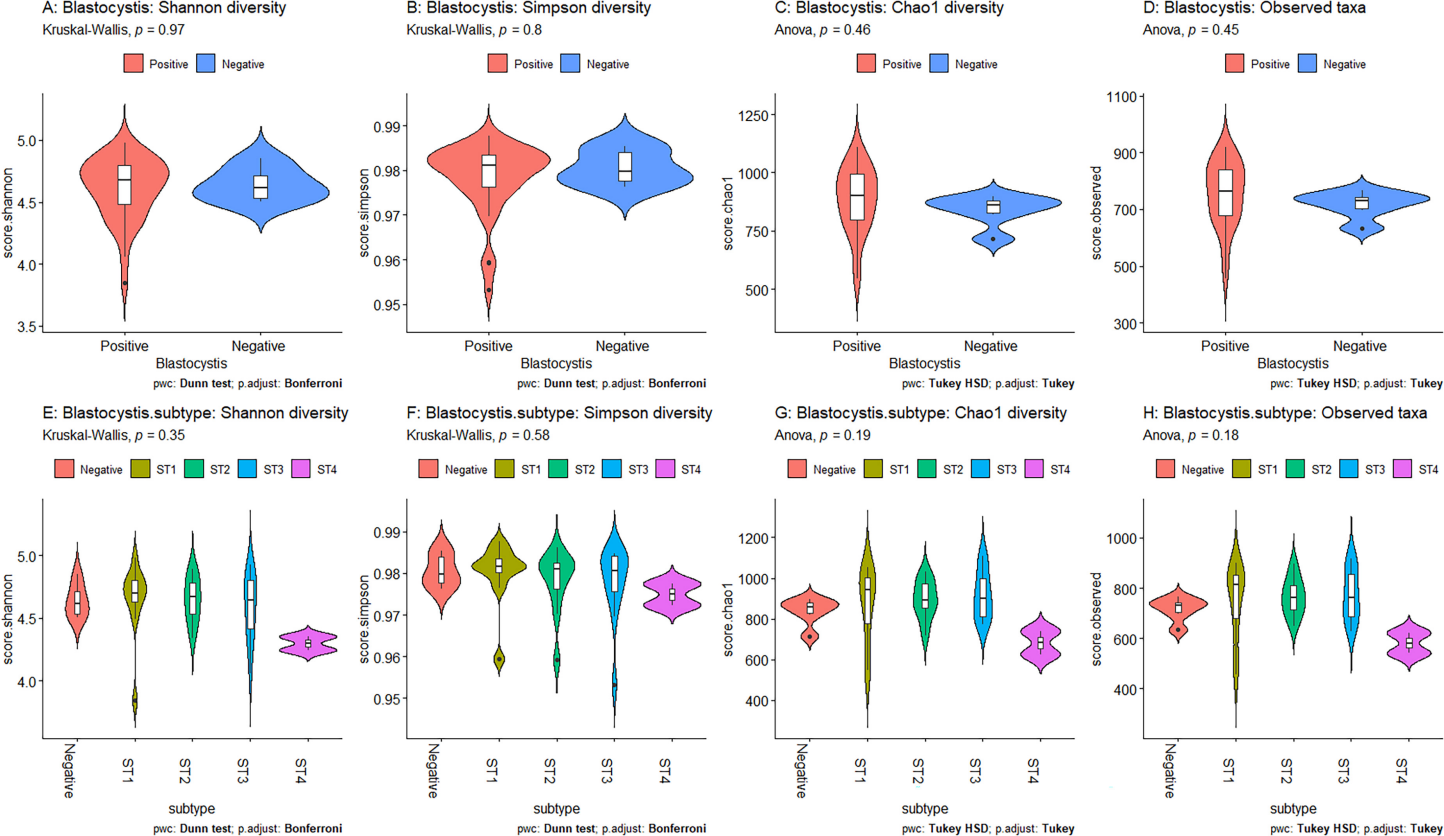
Comparisons of averaged diversity metrics by *Blastocystis* colonization status and subtype. The metrics include Shannon **(A, E)**, Simpson **(B, F)**, Chao1 **(C, G)** and Observed taxa **(D, H)**. Violin plots in panels **A–D** compare *Blastocystis*-positive (red) and *Blastocystis*-negative (blue) samples. Panels **E–H** compare diversity across *Blastocystis* subtypes (colored by subtype). Statistical testing was performed using ANOVA with Tukey’s HSD for normally distributed data and Kruskal-Wallis with Dunn’s test for non-normally distributed data. *P*-values > 0.05 indicate non-significance.

**FIGURE 4:**
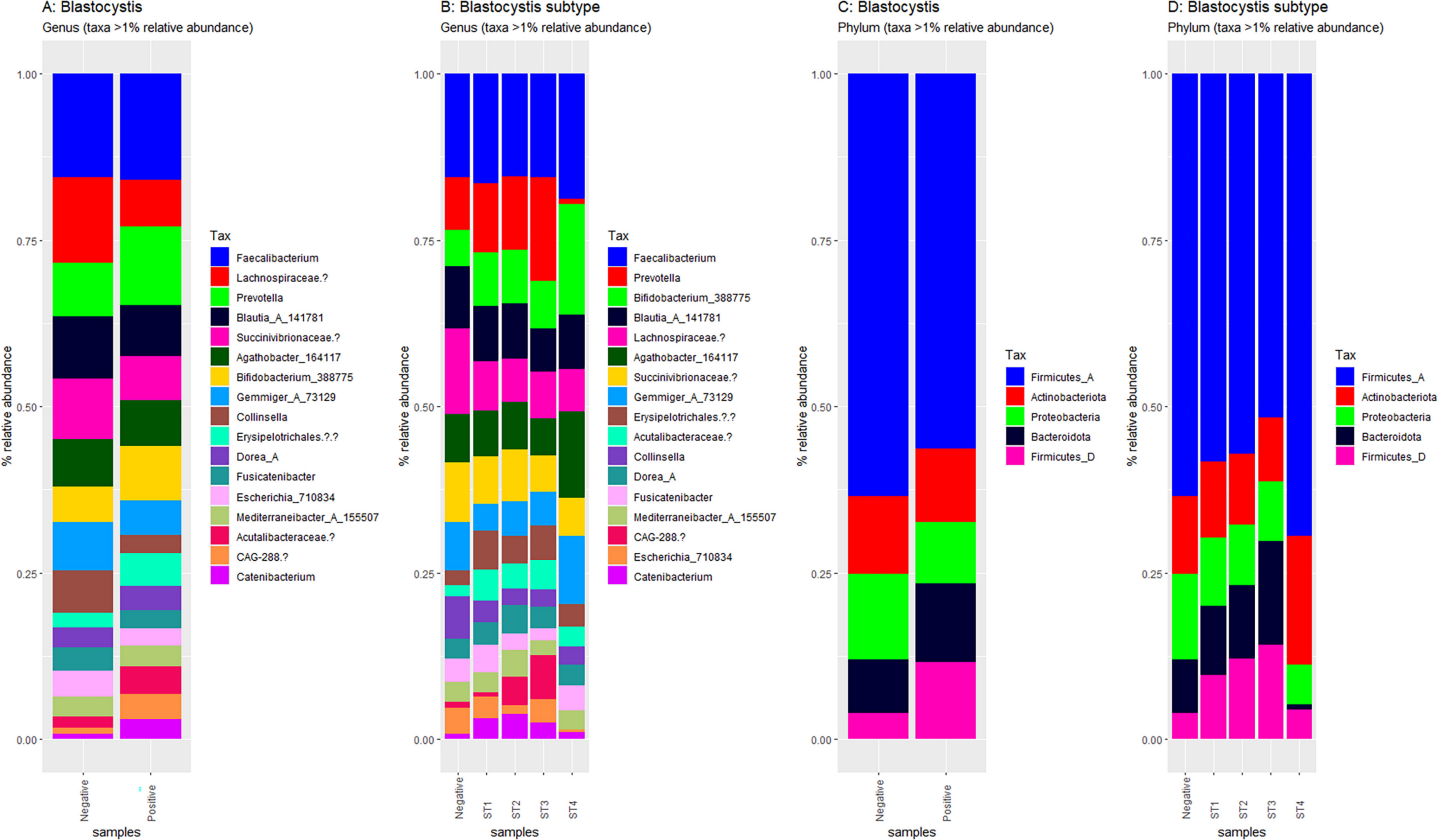
Compositional plots of the most abundant taxa in *Blastocystis*-positive and *Blastocystis*-negative samples. Relative abundances are depicted at the genus level **(A, B)** and phylum level **(C, D)**. Comparisons are by *Blastocystis* presence **(A, C)** and by subtype **(B, D)** Only taxa representing more than 1% of total reads within each sample are displayed.

**FIGURE 5:**
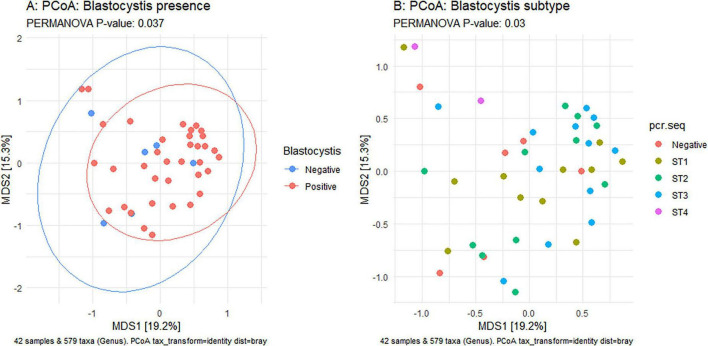
Principal Coordinates Analysis (PCoA) based on Bray-Curtis dissimilarity of samples. Left panel: Comparison of *Blastocystis*-positive (red) and *Blastocystis*-negative (blue) samples. Right panel: comparison of samples by *Blastocystis* subype. Statisitcal differences in group centroids were assessed using PERMANOVA. *P* < 0.05 indicate significance

**FIGURE 6:**
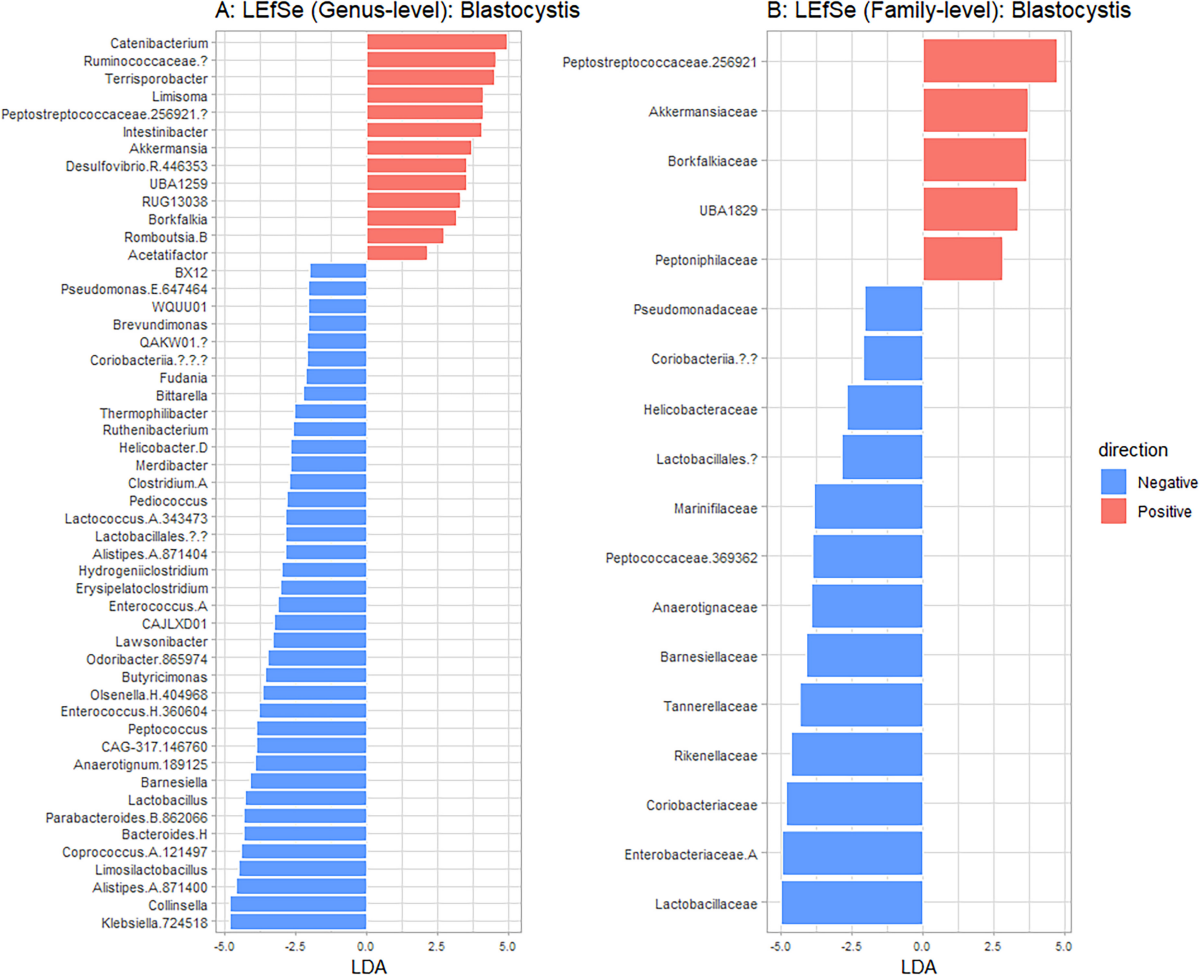
Linear discriminant analysis Effect Size (LEFsE) comparing taxa that discriminate between *Blastocystis* positive (red) and *Blastocystis* negative (blue) samples. Taxa with higher LDA scores indicate stronger discriminative power, with | LDA| > 2 being depicted. Results are shown at genus **(A)** and family **(B)** taxanomic levels.

Using Shannon (Figure 3A), Simpson (Figure 3B), Chao1 (Figure 3C) and observed taxa (Figure 3D), there were no significant differences in bacterial alpha diversity between *Blastocystis* positive and *Blastocystis* negative samples, as confirmed by ANOVA/Kruskal-Wallis tests. The same metrics were then compared at the subtype level (ST1-ST4) (Figure 3E–H). ST1 had the highest average diversity score average across all metrics relative to the *Blastocystis* negative samples, while ST4 samples showed a trend toward lower diversity and richness, but these results were not significant. Comparison of the composition of samples grouped by *Blastocystis* presence is shown in Figure 4 (individual samples are shown in Supplementary Figure 2). At the genus level, *Blastocystis positive* samples had a higher relative abundance of *Prevotella* and *Bifidobacterium* and relatively decreased *Lachnospiraceae* (Figure 4A). When samples were grouped by subtype, ST4 had a notable reduction in *Prevotella* and a marked increase in *Bifidobacterium* along with an increase in *Agathobacter* (Figure 4B). At the phylum level, *Blastocystis positive* samples showed an increase in *Bacteroidota* and a decrease in *Proteobacteria* (Figure 4C). When the samples were grouped by *Blastocystis* subtype, ST4 showed markedly lower relative abundance of *Bacteroidota*, and an increase in Actinobacteriota (Figure 4D). To assess differences in overall bacterial community composition principal coordinate analysis (PCoA) was used based on Bray-Curtis dissimilarities (Figure 5). PERMANOVA testing indicated that the community structure differed significantly between *Blastocystis* positive and *Blastocystis* negative groups, although the effect size was small (*p* = 0.037, R^2^ = 0.046), (Figure 5A). When samples were grouped by *Blastocystis* subtype (Figure 5B), PERMANOVA testing also revealed significant differences in community composition (*p* = 0.03, R^2^ = 0.136), indicating a stronger effect size compared to colonization status alone. Pairwise Adonis was used to assess differences in community composition between groups. A significant difference was observed between ST3 and *Blastocystis* negative samples (Supplementary Table 2). To identify taxa associated with *Blastocystis* colonization status, Linear discriminant analysis Effect Size (LEFsE) was used. Linear discriminant analysis (LDA) scores with an absolute value of 2 were considered indicative of discriminative features. Taxa were aggregated to the genus and family levels (Figure 6). Those with high LDA scores contributed most strongly to distinguishing *Blastocystis* positive from *Blastocystis* negative samples. In total, 38 genera were identified as discriminatory for *Blastocystis* negative samples, while 13 genera were discriminatory for *Blastocystis* positive samples. At the family level, these numbers were 5 and 13, respectively.

## Discussion

The current study presents the first integrative investigation of *Blastocystis* in rural Türkiye, examining human, livestock, and environmental samples to assess its prevalence, subtype distribution, and gut microbiome composition. Anthropometric data from human participants revealed a significant association between BMI and *Blastocystis* colonization in agreement with previous studies (Beghini et al., 2017; Asnicar et al., 2021; Malatyali et al., 2021; Matovelle et al., 2022), with lean individuals having the highest *Blastocystis* positivity rate. A few studies have found a high occurrence of *Blastocystis* in obese individuals (Jinatham et al., 2021; Caudet et al., 2022a,b). Nonetheless, the organism’s presence has been associated with bacterial taxa linked to improved cardiometabolic health profiles (Piperni et al., 2024) and in the obese with lower incidence of metabolic syndrome (Caudet et al., 2022a,b). Moreover, *Blastocystis* colonization was not associated with gut inflammation as indicated by lower fecal calprotectin levels (Nieves-Ramírez et al., 2018). Collectively, these findings support hypotheses of a beneficial role for *Blastocystis* within the gut ecosystem and warrants further exploration to understand its contributions and underlying mechanisms (Deng and Tan, 2025; Stensvold, 2025).

Our results indicate a high occurrence of *Blastocystis* across all studied host species, consistent with findings from other rural communities (El Safadi et al., 2014; Jinatham et al., 2021). However, the true incidence is likely underestimated as several samples negative by microscopy were clearly *Blastocystis* positive, but had to be excluded due to poor sequencing quality. Moreover, several samples positive only by microscopy also failed amplification or sequencing. This was notably the case with the animal and environmental samples. A main issue is the lack of standardization of *Blastocystis* qPCR from environmental samples. Alternative explanations that could account for this, include potential amplification inhibition and mixed infections. Optimizing environmental DNA methodologies are essential and could significantly improve our understanding of ecological reservoirs and transmission pathways.

Overall, 13 subtypes were identified indicating high genetic diversity in the sampled area and associated hosts. Humans predominantly carried subtypes ST1, ST2, and ST3, aligning with global human-associated subtype distributions (Alfellani et al., 2013; Jiménez et al., 2022). Livestock, all of which were ruminants (cattle, sheep, and goats), carried subtypes typical of these animals (Maloney et al., 2019; Rauff-Adedotun et al., 2023; Stensvold et al., 2023; Heydarian et al., 2024; Naguib et al., 2024; Santin et al., 2024). When looking at the community and household level there was barely any sharing of subtypes between humans and their animals. This finding does not seem to match several other studies, which provide evidence that supports the zoonotic transmission of *Blastocystis*. Notably, however, the overall proportion of confirmed zoonotic cases remains low; in nearly all studies, only a small subset of humans shared identical subtypes with animals (Yoshikawa et al., 2009; Rudzińska et al., 2022; Salehi et al., 2022; McCain et al., 2023; Šejnohová et al., 2024). Hence, even though zoonotic transmission is possible, it appears to be infrequent in the studied populations. An alternative explanation might be environmental exposure. *Blastocystis* was previously detected in environmental samples, and recent studies focusing on subtyping have revealed a wide range of subtypes in such samples (Noradilah et al., 2016; Jinatham et al., 2021; Sanhueza Teneo et al., 2025). Herein, we detected *Blastocystis* in water and/or soil samples; however, sequencing failure prevented subtype identification. Collectively, these findings point toward the environment having a role in driving *Blastocystis* transmission. Nonetheless, this route remains comparatively less studied and warrants further investigation.

*Blastocystis* colonization has been associated with distinct gut microbial profiles characterized by increased microbial diversity and bacterial species richness (Audebert et al., 2016; Nieves-Ramírez et al., 2018; Kodio et al., 2019; Even et al., 2021; Piperni et al., 2024; Castañeda et al., 2025). Most of these studies have considered the presence or absence of *Blastocystis*, with comparatively fewer exploring differences across subtypes (Beghini et al., 2017; Tito et al., 2019; Antonetti et al., 2024). Our analyses reveal subtypes specific structuring, indicating that individuals harboring different subtypes have distinct microbial communities consistent with previous findings. In this study, ST4 stood out as it showed a trend toward lower microbial diversity and richness in contrast to earlier studies (Beghini et al., 2017; Tito et al., 2019). Notably, in these studies, nearly all ST4-positive samples (except for two from Asia) originated from westernized populations, mainly from Europe. This suggests that ST4-microbiota associations may vary across populations or environments. Alternatively, the difference could be due to the small number of individuals with ST4 herein. Regardless, this finding highlights the need to investigate diverse populations focusing on *Blastocystis* subtype-level differences.

This study has certain limitations. First, the cross-sectional design precludes assessment of temporal relationships (Tsaousis et al., 2025). While a significant association between BMI and *Blastocystis* carriage was revealed, the test did not establish whether differences among the three BMI groups underlie that finding as other potential confounding factors (e.g., diet, co-existing diseases) were not considered.

## Conclusion

This study not only bridges a knowledge gap by characterizing *Blastocystis* subtype diversity in humans and livestock within a rural Turkish setting but also spearheads a model for integrated One Health investigations in the region. By combining classical parasitological, and microbiome analyses, we demonstrate the feasibility and value of this type of framework. These findings lay the groundwork for future longitudinal and comparative research both within Türkiye and in similar rural communities globally. Additional multidisciplinary collaborative studies will be instrumental in redefining our understanding of intestinal protists–not solely as pathogens, but as complex microbial players in host health and environmental ecosystems (Tsaousis et al., 2024).

## Data Availability

The Blastocystis sequences presented in this study can be found in NCBI under the accession numbers PX444604-PX444713. The raw microbiome data presented in this study can be found under the accession number PRJNA1291457 in the same database.

## Footnotes

1
https://pubmlst.org/bigsdb?db=pubmlst_blastocystis_seqdef&page=sequenceQuery

## Supplementary material

Supplementary Figure 1 Field photographs of the sampling area.

Supplementary Figure 2 *Blastocystis* cells observed in culture.

Supplementary Figure 3 Maximum likelihood phylogeny of *Blastocystis* sequences.

Supplementary Figure 4 Rarefaction curves of 16S sequencing samples. The total number of sequencing reads is shown on the X-axis, and the taxa detected at the corresponding sequencing are displayed on the Y-**axis.**

Supplementary Figure 5 Compositional bar plots showing the most abundant taxa in *Blastocystis*-positive and negative samples. Each bar represents an individual sample. Taxa were aggregated to Genus **(A,B)** and phylum **(C,D)** level. Samples have been grouped by *Blastocystis* colonization status **(A,C)** or *Blastocystis* subtype **(B,D)**. Only taxa representing more than 1% of the total reads within each sample are displayed.
