# Supplementary figures and images for: Blastocystis across humans, animals and the environment in rural Türkiye, and relationships with the human intestinal microbiome
Source: Front Microbiol. 2025 Oct 20;16:1665966. doi: 10.3389/fmicb.2025.1665966 (PMC12580375; doi:10.3389/fmicb.2025.1665966)

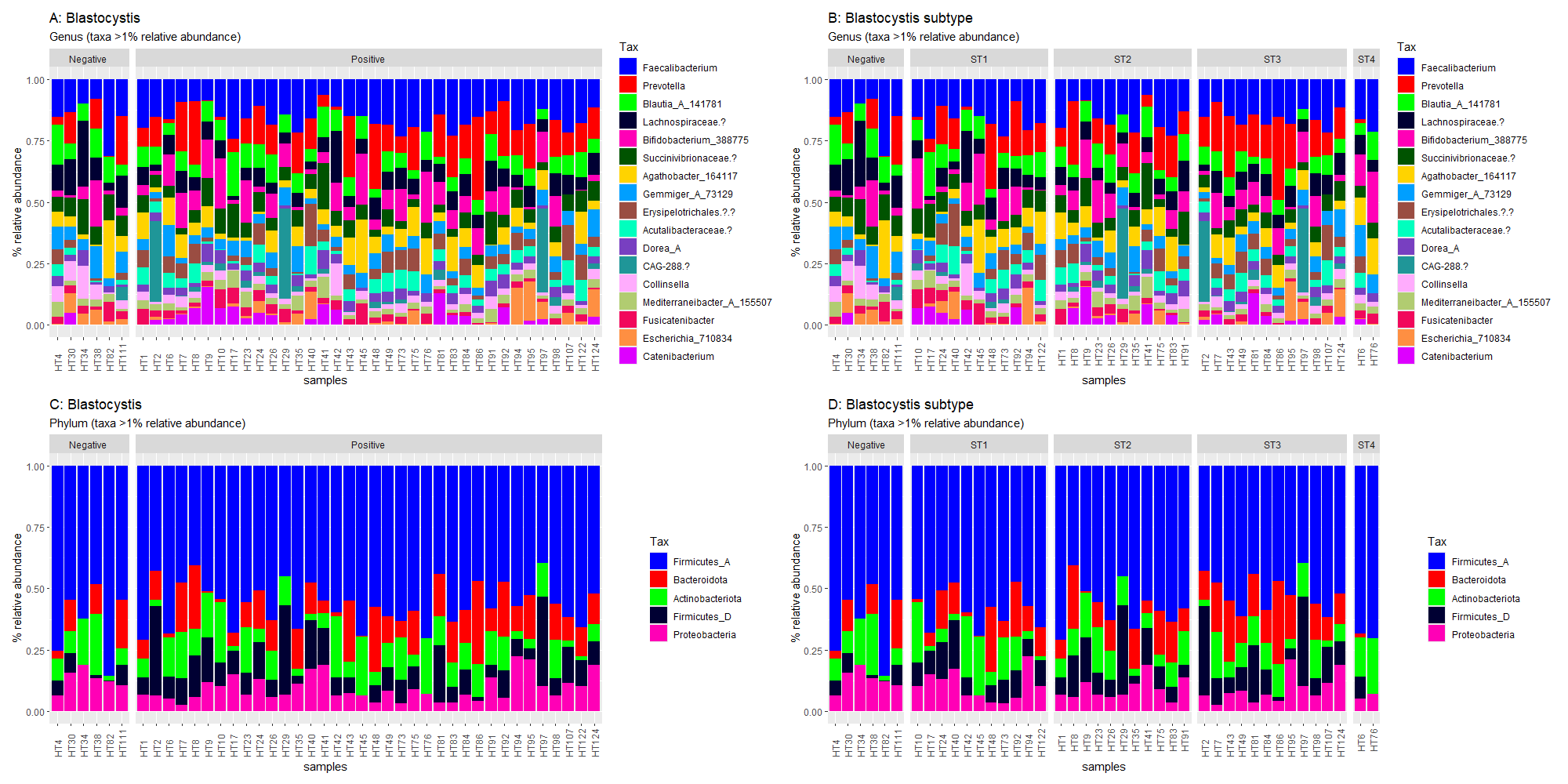

Supplement: Supplementary Figure 1 — Field photographs of the sampling area. [file Image_1.tiff]

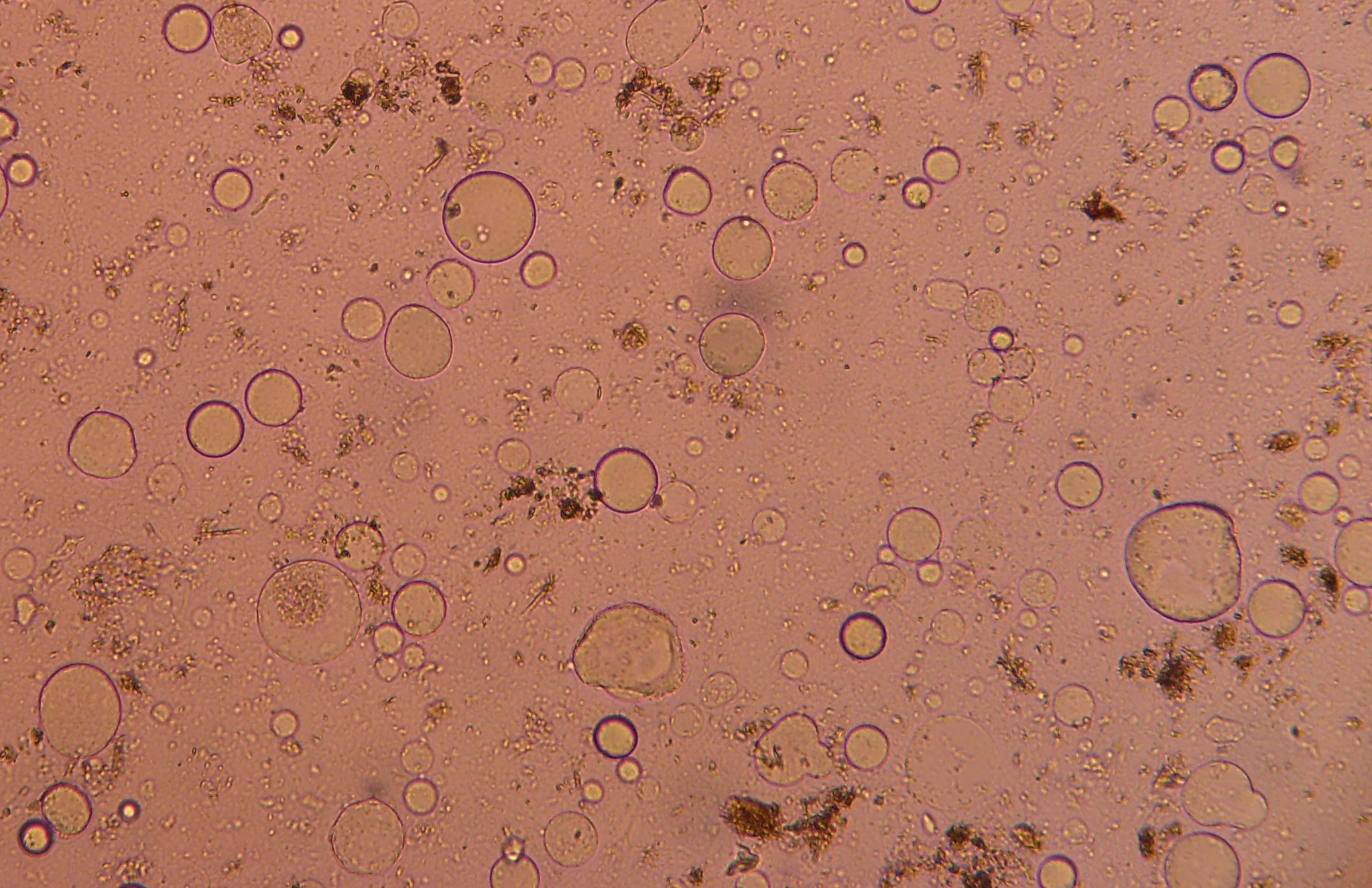

Supplement: Supplementary Figure 2 — Blastocystis cells observed in culture. [file Image_2.jpeg]

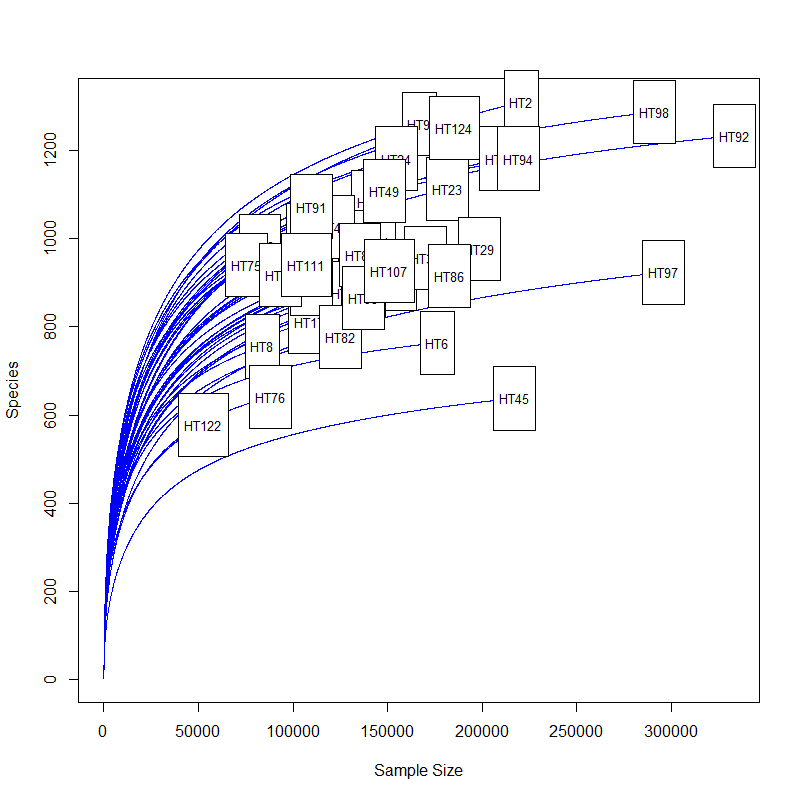

Supplement: Supplementary Figure 3 — Maximum likelihood phylogeny of Blastocystis sequences. [file Image_3.tiff]

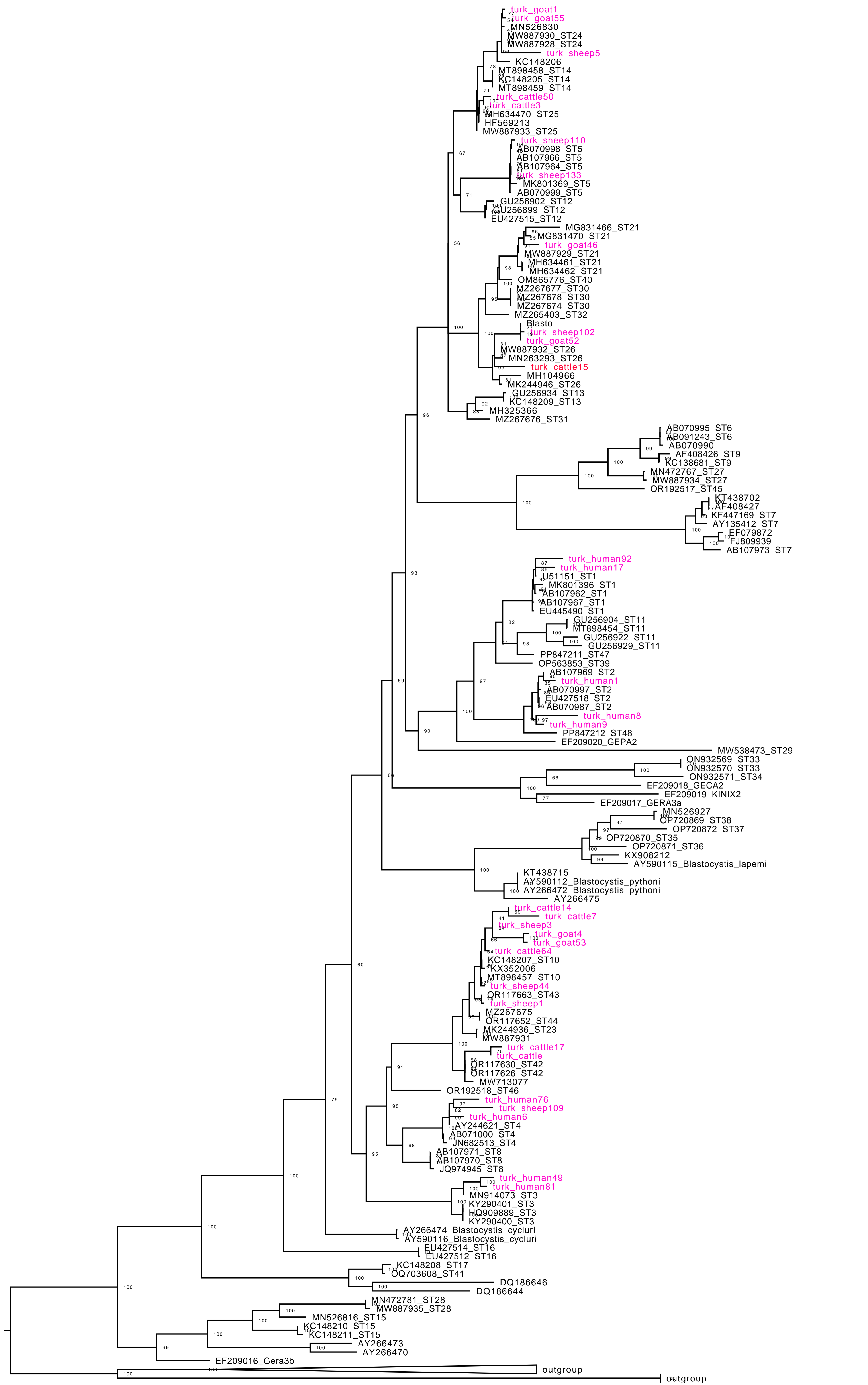

Supplement: Supplementary Figure 4 — Rarefaction curves of 16S sequencing samples. The total number of sequencing reads is shown on the X-axis, and the taxa detected at the corresponding sequencing are displayed on the Y-axis. [file Image_4.pdf]

**
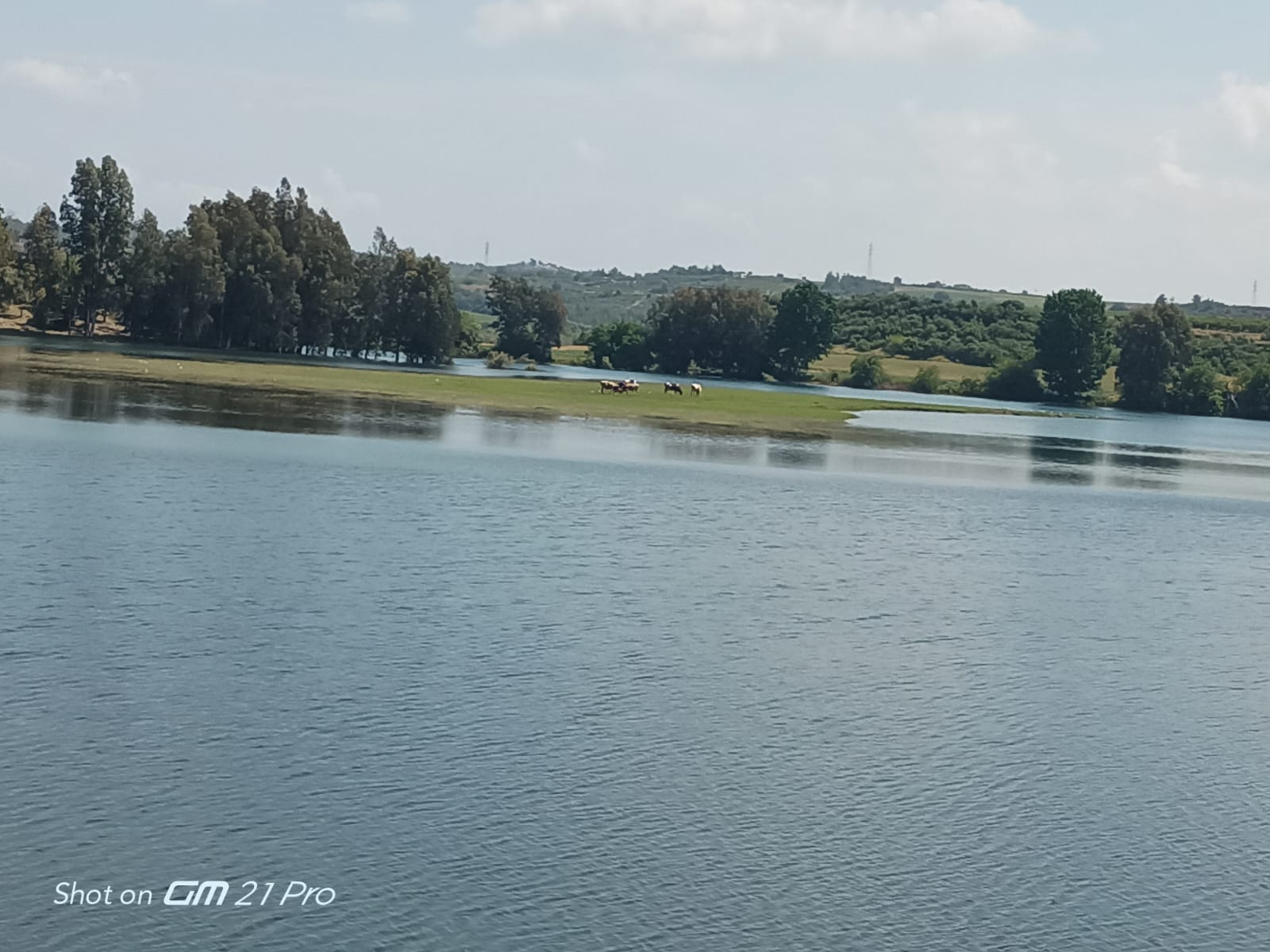
**
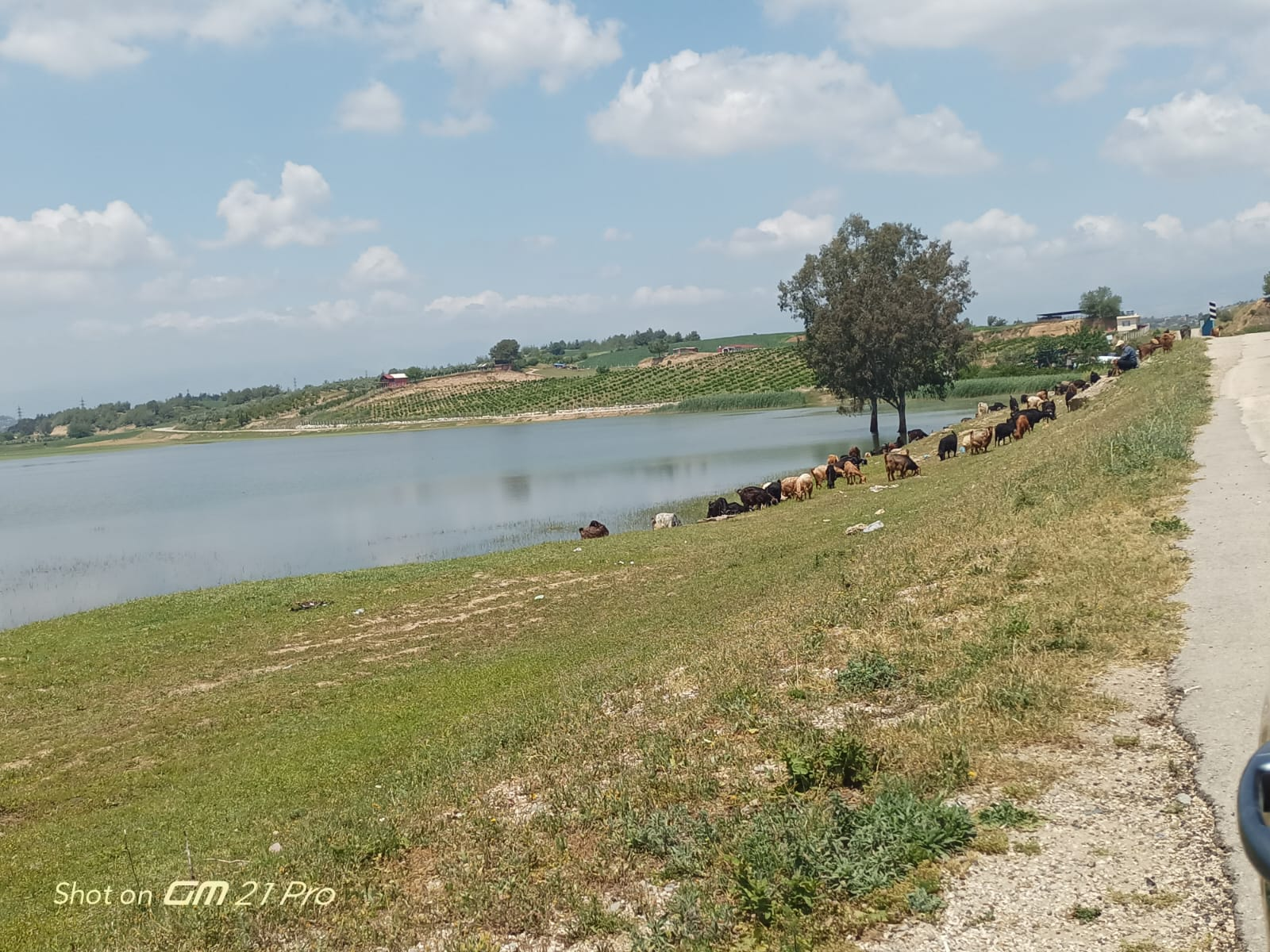

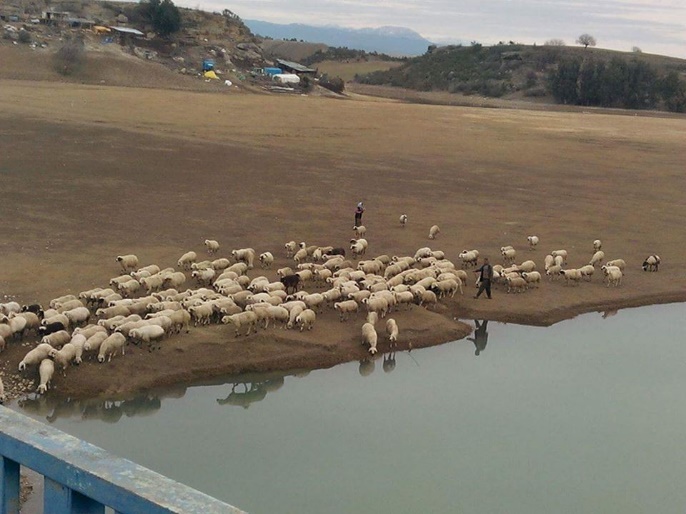

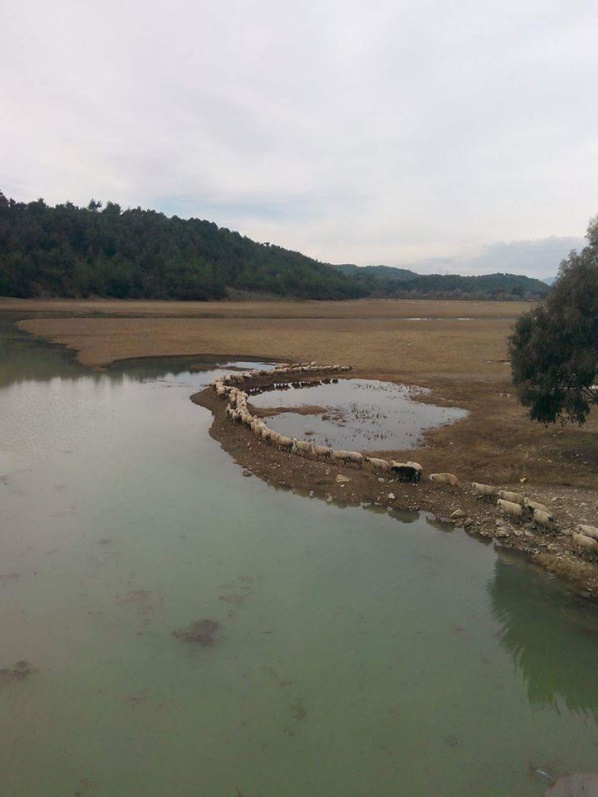

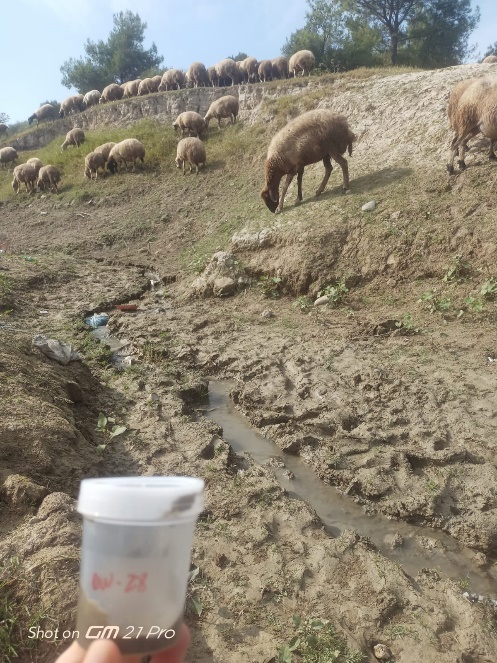

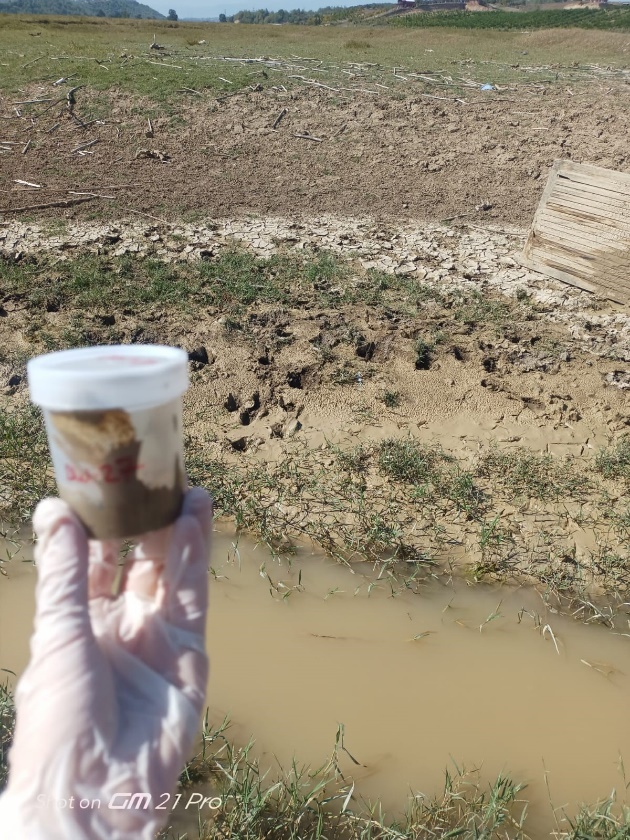
**
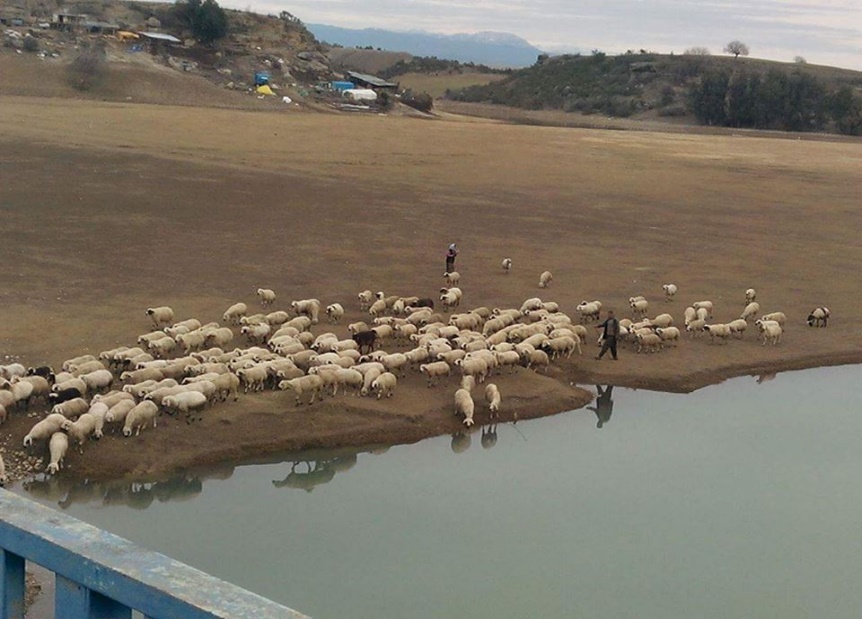
**
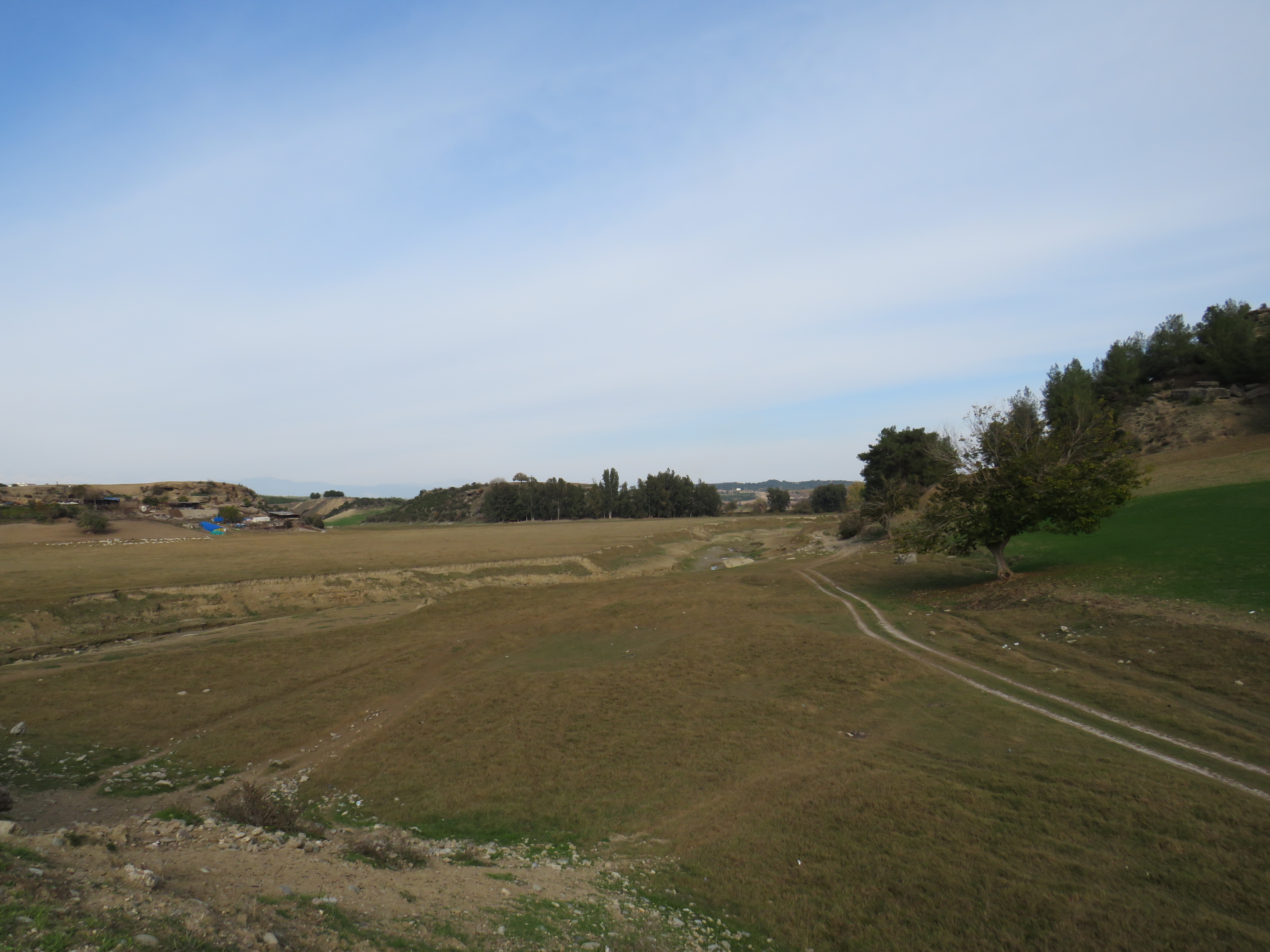

Supplement: Supplementary Figure 5 — Compositional bar plots showing the most abundant taxa in Blastocystis-positive and negative samples. Each bar represents an individual sample. Taxa were aggregated to Genus (A,B) and phylum (C,D) level. Samples have been grouped by Blastocystis colonization status (A,C) or Blastocystis subtype (B,D). Only taxa representing more than 1% of the total reads within each sample are displayed. [file Data_Sheet_1.docx]
