# Supplementary material for: Blastocystis across humans, animals and the environment in rural Türkiye, and relationships with the human intestinal microbiome
Source: Front Microbiol. 2025 Oct 20;16:1665966. doi: 10.3389/fmicb.2025.1665966 (PMC12580375; doi:10.3389/fmicb.2025.1665966)
Supplement: Supplementary file 6 [file Data_Sheet_2.docx]

**Supplementary table 1**: Pairwise Adonis comparisons for each of the Blastocystis subtype statuses and the significance of their comparisons

|  | pairs | Df | SumsOfSqs | F.Model | R2 | p.value | p.adjusted |
| --- | --- | --- | --- | --- | --- | --- | --- |
| 1 | ST2 vs ST3 | 1 | 0.189748 | 1.05176 | 0.047695 | 0.361 | 1 |
| 2 | ST2 vs Negative | 1 | 0.264509 | 1.359521 | 0.083103 | 0.063 | 0.63 |
| 3 | ST2 vs ST4 | 1 | 0.238126 | 1.266667 | 0.103261 | 0.076 | 0.76 |
| 4 | ST2 vs ST1 | 1 | 0.136931 | 0.689495 | 0.033326 | 0.963 | 1 |
| **5** | **ST3 vs Negative** | **1** | **0.331189** | **1.850432** | **0.103663** | **0.004** | **0.04** |
| 6 | ST3 vs ST4 | 1 | 0.27321 | 1.628524 | 0.119494 | 0.058 | 0.58 |
| 7 | ST3 vs ST1 | 1 | 0.191796 | 1.028219 | 0.046677 | 0.403 | 1 |
| 8 | Negative vs ST4 | 1 | 0.246577 | 1.294387 | 0.17745 | 0.121 | 1 |
| 9 | Negative vs ST1 | 1 | 0.266127 | 1.310128 | 0.080326 | 0.063 | 0.63 |
| 10 | ST4 vs ST1 | 1 | 0.244137 | 1.222637 | 0.100031 | 0.127 | 1 |
